# Ginsenosides Are Novel Naturally-Occurring Aryl Hydrocarbon Receptor Ligands

**DOI:** 10.1371/journal.pone.0066258

**Published:** 2013-06-11

**Authors:** Qin Hu, Guochun He, Jing Zhao, Anatoly Soshilov, Michael S. Denison, Aiqian Zhang, Huijun Yin, Domenico Fraccalvieri, Laura Bonati, Qunhui Xie, Bin Zhao

**Affiliations:** 1 Research Center for Eco-Environmental Sciences, Chinese Academy of Sciences, Beijing, China; 2 Department of Environmental Toxicology, University of California, Davis, California, United States of America; 3 Xiyuan Hospital, China Academy of Chinese Medical Sciences, Beijing, China; 4 Department of Earth and Environmental Sciences, University of Milano-Bicocca, Milan, Italy; Philipps University, Germany

## Abstract

The aryl hydrocarbon receptor (AHR) is a ligand-dependent transcription factor that mediates many of the biological and toxicological actions of structurally diverse chemicals. In this study, we examined the ability of a series of ginsenosides extracted from ginseng, a traditional Chinese medicine, to bind to and activate/inhibit the AHR and AHR signal transduction. Utilizing a combination of ligand and DNA binding assays, molecular docking and reporter gene analysis, we demonstrated the ability of selected ginsenosides to directly bind to and activate the guinea pig cytosolic AHR, and to stimulate/inhibit AHR-dependent luciferase gene expression in a recombinant guinea pig cell line. Comparative studies revealed significant species differences in the ability of ginsenosides to stimulate AHR-dependent gene expression in guinea pig, rat, mouse and human cell lines. Not only did selected ginsenosides preferentially activate the AHR from one species and not others, mouse cell line was also significantly less responsive to these chemicals than rat and guinea pig cell lines, but the endogenous gene CYP1A1 could still be inducted in mouse cell line. Overall, the ability of these compounds to stimulate AHR signal transduction demonstrated that these ginsenosides are a new class of naturally occurring AHR agonists.

## Introduction

The aryl hydrocarbon receptor (AHR) is a basic helix–loop–helix PAS-containing transcription factor, which activates gene expression in a ligand-dependent manner [1]. Exposure to 2,3,7,8-tetrachlorodibenzo-p-dioxin (TCDD, dioxin), the prototypical and most potent AHR ligand, results in a wide variety of species- and tissue-specific toxic and biological responses, the majority of which are AHR dependent [2], [3]. Following ligand binding, the cytosolic AHR protein complex, which contains two molecules of hsp90, the X-associated protein 2, and the co-chaperone p23, translocates into the nucleus [4], [5], the ligand-bound AHR is released upon its dimerization with the ARNT (Ah receptor nuclear translocator) protein, and the AHR is converted into its high-affinity DNA binding form [1], [6], [7]. Binding of the heteromeric ligand∶AHR∶Arnt complex to its specific DNA recognition site, the dioxin response element (DRE), upstream of cytochrome P4501A1 (CYP1A1) and other AHR-responsive genes, stimulates their transcription [1], [3].

The best characterized high-affinity ligands for the AHR include a variety of synthetic halogenated aromatic hydrocarbons (HAHs), such as the polychlorinated dibenzo-p-dioxin, dibenzofurans, and biphenyls, as well as numerous polycyclic aromatic hydrocarbons (PAHs), such as benzo(a)pyrene, 3-methylcholanthrene, and others [2], [8]. More recently, a relatively large number of natural and synthetic AHR ligands (agonists and antagonists) whose structures and physicochemical characteristics are dramatically different from that of the prototypical HAH and PAH have been identified and characterized [9]–[11]. While the relative potencies of these diverse ligands in intact cells and animals are typically much lower than that of the HAHs and PAHs, predominantly due to differences in their affinity, intrinsic efficacy, and metabolic stability [8], [10]–[12], these results demonstrate that the AHR has an extremely promiscuous ligand binding pocket, and raised questions as to the actual spectrum of chemicals that can bind to and activate the AHR and AHR signaling pathway. Accordingly, we have carried out bioassay screening analysis of a wide variety of natural compounds and extracts with the goal to identify and characterize novel AHR ligands, and extend our understanding of the AHR ligand structural diversity.

Ginseng has been used as traditional medicine in China, Korea, Japan and other Asian countries for thousands of years. While there are seven major species of ginseng in East Asia, Central Asia, and North America, most studies have focused on constituents from three common species: *Panax ginseng* (Asian ginseng), *Panax quinquefolius* (American ginseng), and *Panax japonicus* (Japanese ginseng). The majority of the diverse pharmacological and biochemical actions of ginseng appeared to be attributed to ginseng saponins (ginsenosides), and more than 60 different ginsenosides have been isolated from members of the Panax genus [13]. While there is was antagonistic action by the ginseng saponin components resulting in inhibition of cellular proliferation, ginsenosides can also stimulate cell growth [14].

The diversity of AHR ligand structure coupled with the ability of numerous natural products to bind to the AHR [8]–[11], and the recent identification of two common clinically used ginsenosides (Rg_1_ and Rb_1_) that can increase CYP1A1 mRNA levels in human cells in culture [15], suggests that these compounds may be AHR ligands. However, while induction of human CYP1A1 gene expression is known to be mediated by the AHR, several studies have also demonstrated induction by the retinoic acid receptor and other signaling mechanisms [3], [16]–[18]. Additionally, since the study of Wang et al. (2008) did not determine whether these compounds directly stimulated induction of CYP1A1, or whether the response was secondary (i.e. due to a ginsenoside metabolite or activation of an alternative pathway), the mechanism(s) responsible for ginsenoside-dependent induction of CYP1A1 still remains an open question [15]. Accordingly, here we described the results of studies examining the ability of a series of ginsenosides to stimulate AHR-dependent gene expression and confirmed their identity as AHR ligands.

## Materials and Methods

### Chemicals

**Figure 1:**
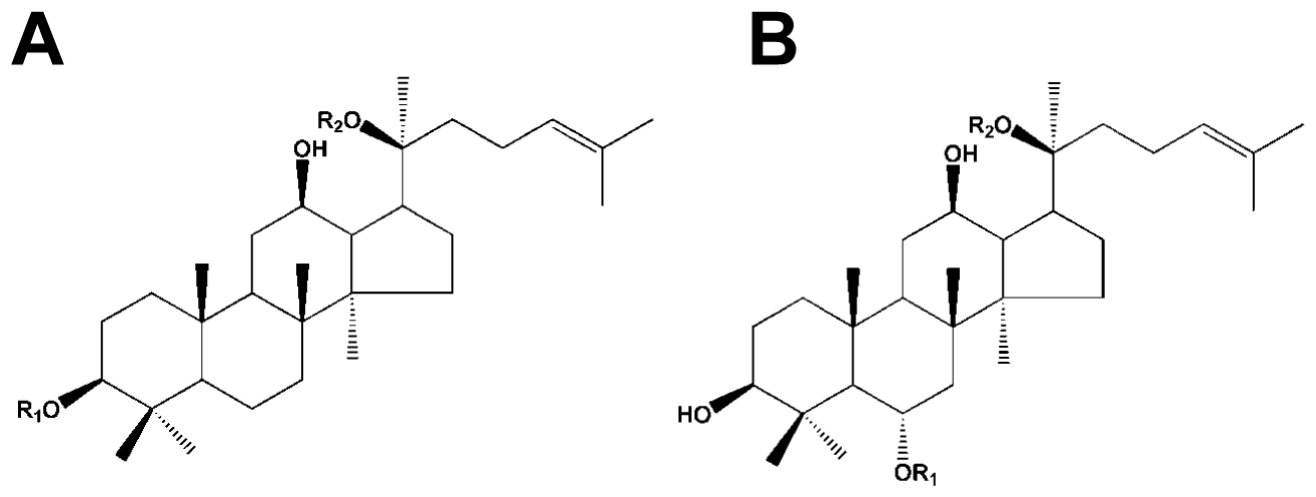
Backbone structures of ginsenosides. Each type of ginsenosides differ at two side chains OR_1_ and OR_2_ attached to the common steroid ring.

The specific ginsenosides used in this study (Rb_1_, Rb_2_, Rb_3_, Rc, Rd, Re, Rg_1_, Rg_2_, Rh_1_, Rh_2_, PPD, PPT, F11, and a total ginsenosides (TG) mixture (Figure 1 & Table 1), were kindly provided by Dr. Huijun Yin (Chinese Academy of Medical Sciences, Beijing, China) and were of greater than 98% purity. TCDD, 2,3,7,8-tetrachlorodibenzofuran (TCDF), and [^3^H]-TCDD (10 Ci/mmol) were obtained from S. Safe (Texas A&M University, College Station, TX USA). [^32^P]-ATP (6000 Ci/mmol) was purchased from Amersham (Arlington Heights, IL USA) and DMSO from Sigma-Aldrich. Cell culture media was purchased from Gibco (Invitrogen), fetal calf serum was purchased from Lonza (BioWhittaker) and G418 was from Gemini Bio-Products (Woodland, CA USA). Water was purified using a Milli-Q water purification system (Millipore). All other chemicals were of analytic purity.

**Table 1:** Structures of ginsenosides examined in this study.

Category	Name	Side chain	
		R1	R2
PPD type	Rb1	-Glc2-Glc	-Glc6-Glc
	Rb2	-Glc2-Glc	-Glc6-Ara(p)
	Rb3	-Glc2-Glc	-Glc6-Xyl
	Rc	-Glc2-Glc	-Glc6-Ara(f)
	Rd	-Glc2-Glc	-Glc
	Rh2	-Glc	-H
	PPD	-H	-H
PPT type	Re	-Glc2-Rha	-Glc
	Rg1	-Glc	-Glc
	Rg2	-Glc2-Rha	-H
	Rh1	-Glc	-H
	PPT	-H	-H
Special type	F11		
	TG		

Abbreviations for carbohydrates are as follows: Glc, glucopyranoside; Ara, arabinopyranoside; Rha, rhamnopyranoside; Xyl, xylopyranoside; TG, total ginsenosides (mixed compounds). Superscripts indicated the carbon in the glucose ring that linked the two carbohydrates.

### Cell Culture, Chemical Treatment, and Ahr-Dependent Luciferase Reporter Gene Expression

Recombinant guinea pig intestinal adenocarcinoma (G16L1.1c8) cells and rat (H4L1.1c4), mouse (H1L1.1c2) and human (HG2L6.1c3) hepatoma cells were grown and maintained as described [19]. G16L1.1c8, H4L1.1c4 and H1L1.1c2 cells contain the stably integrated DRE-driven firefly luciferase reporter plasmid pGudLuc1.1, HG2L6.1c3 cells contain pGudLuc6.1 [20] and the transcriptional activation of those plasmids occurs in a time-, ligand-, dose-, and AHR-dependent manner [19], [21]. Cells were plated into white, clear-bottomed 96-well tissue culture dishes at 75,000 cells per well and allowed to attach for 24 h. Cells were incubated with carrier solvent DMSO (1% final solvent concentration), TCDD (1 nM), or the indicated ginsenoside (for measurement of agonist activity), or 1 nM TCDD plus the indicated ginsenoside (for measurement of antagonist activity) for 4 h at 37°C. For luciferase measurement, sample wells were washed twice with phosphate-buffered saline, followed by the addition of cell lysis buffer (Promega) and shaking of the plates for 20 min at room temperature to allow cell lysis. Measurement of luciferase activity in each well was carried out using a microplate luminometer (TECAN Infinite 200 Multi Reader)with automatic injection of Promega stabilized luciferase reagent. Luciferase activity in each well was expressed relative to that induced by 1 nM TCDD.

### Homology Modeling

The LBD structure of the guinea pig AHR (gpAHR) was predicted by homology modeling, using the same procedure previously adopted for the mouse, rat and human AHRs (mAHR, rtAHR, huAHR) [22]. In brief, three X-ray structures of HIF-2á co-crystallized with the THS ligands (3F1O, 3H7W and 3H82) were used as templates. MODELLER version 9v7 [23]–[25] was used to perform homology modeling, by activating the option to transfer all the THS structures from the templates to the final homology model. One hundred models were obtained by random generation of the starting structure and the DOPE score [26] was used to rank the models. Four conformational clusters were identified in this set of models, and a representative conformation for each cluster (the one with the best DOPE score) was selected for the ensemble docking calculations.

### Ligand Conformational Analysis

Conformational search of the ginsenoside ligands (PPD and PPT) in the free state was performed using the MacroModel 9v9 [27] program included in Maestro 9v3 [28] with the following parameters: OPLS-2005 force field [29], implicit (Generalized Born/Solvent Accessible, GB/SA) water solvation [30], automatic set up of the conformational degrees of freedom, and Monte Carlo Multiple Minimum (MC/MM) random search algorithm. The five most stable conformational minima for each ligand were selected for docking calculations.

### Molecular Docking

The ensemble docking approach previously proposed for ligand docking to the AHR homology models [22] was used. Accordingly, the four representative conformations selected in the ensemble of homology models of the gpAHR were used for docking. To account for the ginsenosides' conformational variability, the five conformations selected for PPD and PPT were utilized for docking analysis. Flexible ligand docking of the two ginsenosides and the TCDD was carried out using the Glide 5.8 program [31] with the Glide extra-precision (XP) protocol [32]. The binding box was centered in the averaged Cartesian coordinates of the template THS ligands centroids, with 25 Å sides length. All the other parameters were of the default ones. The final best scoring pose for each ligand was selected by using the Glide XP scoring function [32]. Docking calculations with the same ligands were performed, for comparative purposes, in the modeled mAHR, rtAHR and huAHR LBDs [22] using the same computational protocol.

### Refinement of The Docking Poses

Energy minimization of all the obtained AHR/ligand complexes was carried out with the MacroModel 9.9 program [27], [28]. The OPLS-2005 force field [29], the implicit GB/SA water solvation model [30] and the TNCG minimization algorithm were employed in this analysis. Different degrees of system flexibility were imposed: the ligands and the side chains of the residue shell within 5 Å from the ligands were defined as free to move; the backbones of the residue shell within 5 Å from the ligands were constrained with a force constant of 200 kJ*mol^−1^*Å^−2^, the residues within 5–7 Å from the ligands were constrained with a force constant of 500 kJ*mol^−1^*Å^−2^, and all the remaining residues were frozen.

### Rt-Pcr Analysis of Endogenous Gene Cyp1a1 Induction

Forward and reverse RT-PCR primers were synthesized and contained the following sequences: mCYP1A1 FP, 5′-CCTCATGTACCTGGTAACCA-3′; and mCYP1A1 RP, 5′-AAGGATGAATGCCGGAAGGT-3′, and a highly conserved region of a constitutively expressed housekeeping gene, GAPDH FP, 5′-TGCACCACCAACTGCTTAG-3′; and GAPDH RP, 5′-GATGCAGGGATGATGTTC-3′ [33]. Confluent mouse hepatoma cells (hepa1c1c7) were treated with 1% carrier solvent (DMSO), 1 nM TCDD, or 10 µM ginsenoside for 4 h, respectively, prior to mRNA isolation using TRIzol (Invitrogen). Single stranded cDNA was synthesized using RevertAid First Strand cDNA Synthesis Kit (Fermentas) according to the manufacturer's instructions and used for PCR amplification. PCR reactions were conducted in final volume of 20 µL and performed on equal amounts of reverse-transcribed products, using SYBR Green Master mix and Rox reference dye, according to the manufacturer's instructions (Promega). All PCR reactions were performed and analyzed in a Stratagene real-time PCR machine (MX3005P, USA). The specificity of amplification was confirmed by melting curves and by gel electrophoresis.

### Preparation of Cytosol Extracts

Male Hartley guinea pigs (250–300 g), obtained from Charles River Breeding Laboratories (Wilmington, DE), were exposed to 12 h of light and 12 h of dark daily and were allowed free access to food and water. Hepatic cytosol was prepared in HEDG buffer (25 mM Hepes (pH 7.5), 1 mM EDTA, 1 mM DTT, and 10% (v/v) glycerol) as previously described [21]. The resulting cytosolic extract was stored frozen at −80°C until use. Protein concentrations were determined by dye binding using bovine serum albumin as the standard. All procedures and experiments with animals and animal-derived materials were reviewed and approved by the University of California Davis Institutional Animal Care and Use Committee (approval ID 08-13392).

### Gel Retardation Assay

Complementary synthetic oligonucleotides containing the DRE3 AHR DNA binding site 5′-GATCTGGCTCTTCTCACGCAACTCCG-3′ and 5′-GATCCGGAGTTGCGTGAGAA GAGCCA-3′ were prepared, annealed, and end-labeled with [^32^P]ATP as described [21]. Guinea pig hepatic cytosol (8 mg/ml in HEDG) was incubated for 2 h in a room temperature water bath with DMSO (2%), TCDD (20 nM)), or the indicated ginsenoside (200 µM). An aliquot of the reaction was mixed with poly[dI•dC] and [^32^P]-DRE (100,000 cpm), and AHR∶DRE∶[^32^P]DRE complexes were resolved by gel retardation analysis, visualized by autoradiography and quantified by phosphorimager analysis (Molecular Dynamics, Sunnyvale, CA USA) of the dried gels [21].

### Ligand Binding Analysis

Aliquots of guinea pig hepatic cytosol (2 mg protein/ml) were incubated with 2 nM [^3^H]TCDD in the presence of DMSO (1%), TCDF (200 nM), the indicated solvent or ginsenoside (200 µM) for 2 h in a room temperature water bath. [^3^H]TCDD binding in aliquots of the incubation (200 µl) was determined by HAP binding as previously described [21]. The total amount of [^3^H]TCDD specific binding was obtained by subtracting the nonspecific binding ([^3^H]TCDD + TCDF) from the total binding ([^3^H]TCDD), and the ability of ginsenosides to bind to the AHR was indicated by their ability to competitively reduce [^3^H]TCDD specific binding.

## Results

### Agonist And Antagonist Activity of Ginsenosides in Guinea Pig G16l1.1c8 Cells

**Figure 2:**
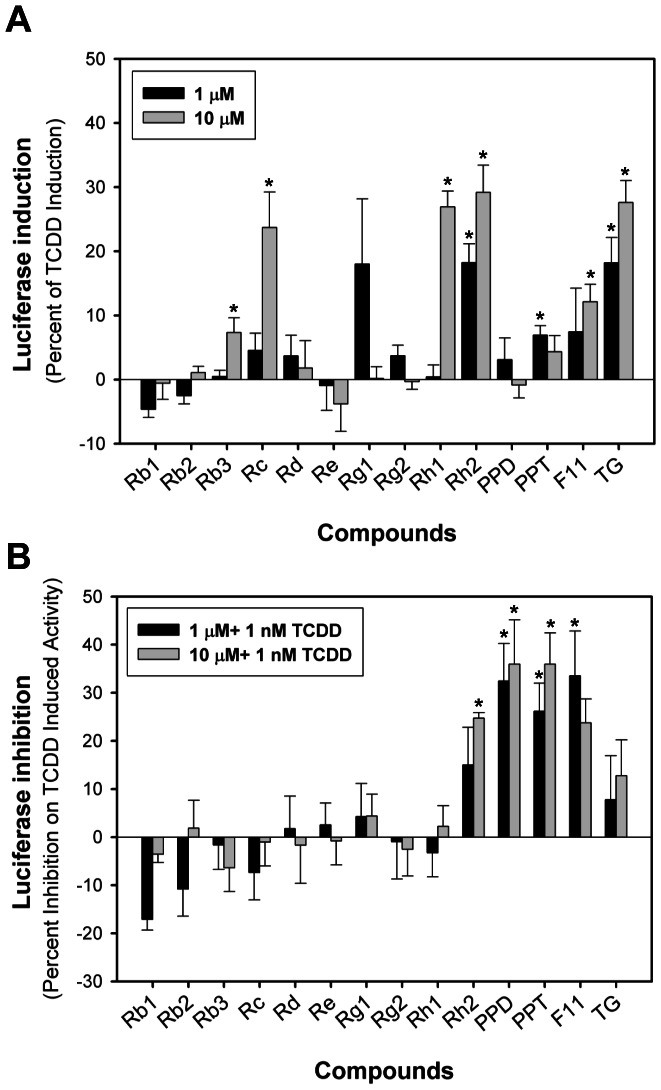
Induction of luciferase activity by ginsenosides in AHR-responsive recombinant guinea pig G16L1.1c8 cells. (A) G16L1.1c8 cells were treated with 1 µM or 10 µM ginsenosides for 4 h. (B) G16L1.1c8 cells were treated with 1 µM/10 µM ginsenosides + 1 nM TCDD for 4 h and luciferase activity was determined as described in Materials and Methods section. Values were expressed in the figure as the percentage of maximal TCDD induction and represented the mean ± SD of triplicate determinations. The asterisk indicated that the values of induction or inhibition was significantly increased compared to DMSO-treated sample at p<0.05 (*).

We first examined the AHR agonist activity of a series of ginsenosides by testing their ability to stimulate AHR-dependent reporter gene expression in recombinant guinea pig intestinal adenocarcinoma cells (G16L1.1c8) that contain the stably transfected DRE-luciferase reporter plasmid pGudLuc1.1. Concentration-dependent induction of luciferase by ginsenosides at 4 h was observed in this cell line, and some ginsenosides were found to stimulate AHR-dependent reporter gene expression at concentrations of 1 µM (Rh_2_, PPT) and 10 µM (Rb_3_, Rc, Rh_1_, Rh_2_, F11, TG) (Figure 2). Both PPD and PPT reduced TCDD-dependent luciferase induction in the same cell line, and luciferase gene expression induced by TCDD was reduced by Rh_2_, PPD, PPT and F11 in guinea pig cell line.

### Stimulation of AHR transformation and DNA binding by TCDD and ginsenosides *in vitro*

**Figure 3:**
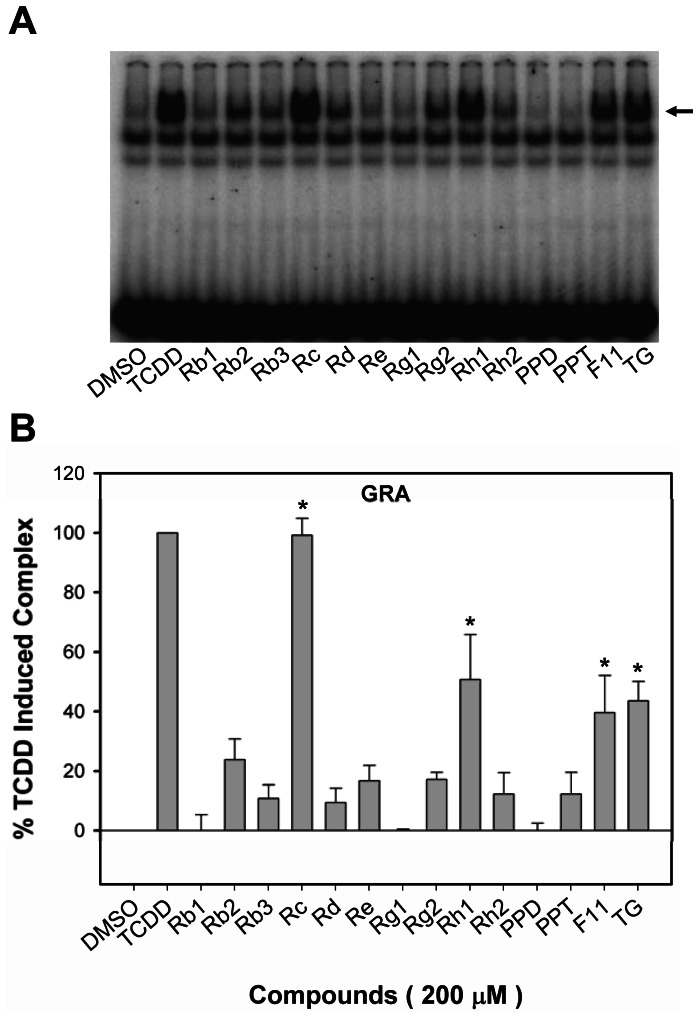
Stimulation of AHR transformation and DNA binding by TCDD and ginsenosides *in vitro*. Guinea pig hepatic cytosol (8 mg protein/mL) was incubated with DMSO (20 µL/mL, final concentration), 20 nM TCDD, or 200 µM of the indicated ginsenosides for 2 h at 20°C. Protein-DNA complexes were resolved by gel retardation analysis. Protein–DNA complexes were resolved by gel retardation analysis (a) and the amount of induced protein–DNA complex formation determined by phosphorimager analysis (b). The arrow indicated the position of the AHR∶DRE complex. Values were expressed in the figure as the percentage of maximal combination by TCDD and represented the mean ± SD of triplicate determinations. The asterisk indicated that the combination was significantly induced compared to DMSO-treated sample at p<0.05 (*). Induced complex formation at all concentrations of TCDD ≥10^−11^ M and of ginsenosides ≥10^−7^ M were significantly greater than the DMSO-treated sample at *p<*0.01 as determined by Student's *t*-test.

The reporter gene expression results demonstrated the ability of selected ginsengosides to activate AHR-dependent gene expression, but they did not address whether this induction was direct (i.e. the ginsenoside binds to and activates the AHR) or indirect (a metabolite of the ginsenoside binds to the AHR and/or it activates the AHR by a mechanism that does not involve direct binding to the AHR). To address this, we examined the ability of ginsenosides to directly stimulate transformation and DNA binding of guinea pig cytosolic AHR *in vitro* using gel retardation analysis. The results of these analyses (Figure 3) revealed that the ginsenosides Rc, Rh_1_, F11 and TG could stimulate AHR∶DRE complex formation to a level greater than 40% of that maximally induced by TCDD, with Rc producing maximal AHR∶DRE complex formation (99±6 % of that of TCDD). While the DNA binding analysis results indicated that at least four ginsenosides could stimulate AHR transformation and DNA binding *in vitro*, we did not examine whether these compounds were actually AHR ligands.

### Competitive Binding of Ginsenosides to The Guinea Pig Hepatic Cytosolic Ahr

To determine whether these ginsenosides were ligands for the AHR, we evaluated their ability to compete with [^3^H]TCDD for binding to the AHR. The results of these studies revealed that many ginsenosides (Table 2), including Rc, Rd, Re, Rg_2_, Rh_1_, Rh_2_, PPD, PPT, F11 and TG, could competitively bind to the AHR, displacing between 20 and 80% of [^3^H]TCDD specific binding. Similar to its relatively high efficacy to stimulate AHR DNA binding, Rc competitively displaced ∼90% of [^3^H]TCDD specific binding. While competitive binding by Rc, Rh_1_, Rh_2_, F11 and TG were consistent with their agonist activities in the gene expression studies, comparison of binding and gene expression data (Figure 2) suggested that these compounds were partial agonists. In contrast, the competitive binding and inhibition of TCDD-inducible gene expression observed with PPD and PPT would suggest that they functioned as AHR antagonists. Interestingly, Re appeared to be a unique nonproductive AHR ligand for guinea pig in that it could effectively compete for ligand binding but not significantly stimulate or inhibit AHR-dependent gene expression, but it could induce AHR-dependent luciferase expression in rat cells (Table 3).

**Table 2:** Competitive binding of ginsenosides to the guinea pig hepatic cytosolic AHR.

Competitor	Concentration (µM)	[^3^H]-TCDD Specific Binding (Percent of Displacement)^a^
Rg1	200	6.1±7.6
Rb1	200	14.7±7.4
Rg2	200	21.2±8.3b
Rd	200	22.3±6.2b
Rb2	200	23.5±17.4
Rb3	200	23.7±14.7
F11	200	28.0±9.1b
TG	200	31.0±1.5b
Re	200	38.6±12.0b
PPD	200	45.5±3.7b
Rh1	200	45.9±23.2b
PPT	200	48.1±3.5b
Rh2	200	50.9±4.4b
Rc	200	88.2±3.9b

Guinea pig hepatic cytosol was incubated with 2 nM [^3^H]-TCDD in the absence or presence of 200 nM TCDF or the indicated ginsenoside for 2 hours at 20°C and [^3^H]-TCDD specific binding was determined using the hydroxyapatite binding assay as described in the Materials and Methods section. ^a^ Values were expressed as a percent of the total [^3^H]-TCDD specific binding and represented the mean ± SD of triplicate determinations. ^b^ Values represented that the amount of [^3^H]-TCDD specific binding was significantly displaced by the competitors at p<0.05 (*) as determined by Student's *t*-test.

**Table 3:** Ginsenoside agonist activity in AHR-responsive recombinant guinea pig (G16L1.1c8), rat (H4L1.1c4), mouse (H1L1.1c2) and human (HG2L6.1c3) cells.

Treatment		Luciferase Activity in Different Cell Lines (Percent of TCDD)^a^			
Chemical	Concentration	G16L1.1c8	H4L1.1c4	H1L1.1c2	HG2L6.1c3
TCDD	1 nM	100±12	100±11	100±16	100±3
Rb_1_	10 µM	1±2	−1±0	−1±1	3±2
Rb_2_	10 µM	1±1	−2±1	−2±0	19±4^b^
Rb_3_	10 µM	7±2^b^	13±3^b^	5±1l	5±2
Rc	10 µM	24±6^b^	23±6^b^	17±3^b^	19±2^b^
Rd	10 µM	2±4	0±0	−2±1	5±1
Re	10 µM	0±3	38±13^b^	4±2	1±1
Rg_1_	10 µM	0±2	12±0^b^	10±3^b^	1±1
Rg_2_	10 µM	0±1	2±1	5±2	2±0
Rh_1_	10 µM	27±2^b^	5±4	2±2	6±0
Rh_2_	10 µM	29±4^b^	0±1	3±4	8±2^b^
PPD	10 µM	0±2	−3±1	4±2	5±2
PPT	10 µM	4±2	6±3	7±1^b^	6±1
F11	10 µM	12±3^b^	8±2^b^	3±4	6±1
TG	10 µM	28±3^b^	3±2	2±3	7±2^b^

Cells were incubated with 10 µM of indicated ginsenoside for 4 hours and luciferase activity was determined as described in Materials and Methods section. ^a^ Values were expressed as the percentage of 1 nM TCDD induction and represented the mean ± SD of triplicate determinations. ^b^ Values were significantly different from the DMSO-treated sample at p<0.05 as determined by Student's *t*-test.

### Endogenous Gene Cyp1a1 Expression

**Figure 4:**
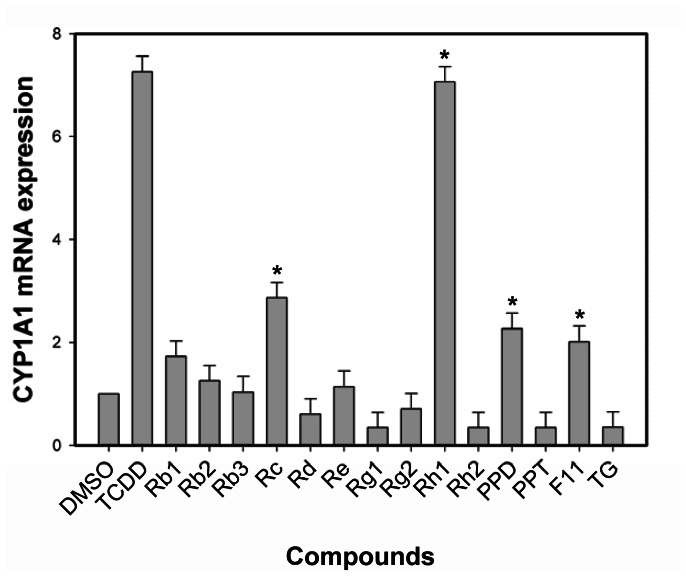
Endogenous gene CYP1A1 expression in mouse hepatoma cells. Hepa1c1c7 cells were incubated with DMSO (1%, final concentration), TCDD (1 nM), or ginsenosides (10 µM) for 4 h at 37°C, mRNA was extracted, subjected to RT-PCR and amplification. The asterisk indicated that the gene expression was significantly induced compared to DMSO-treated sample at p<0.05 (*).

To confirm the ability of ginsenosides to induce expression of an endogenous AhR-responsive gene, in addition to the stably transfected DRE-luciferase reporter, we examined its effect on CYP1A1 expression (i.e., mRNA levels) using RT-PCR. Incubation of mouse hepatoma (hepa1c1c7) cells with ginsenosides (Rc, Rh1, PPD, F11) for 4 h increased CYP1A1 mRNA levels, albeit to a lower level than that induced by TCDD (Figure 4), but these data were consistent with the reporter gene induction results and the AhR agonist activity of these ginsenosides.

### Molecular Docking on Homology Models

To analyze the molecular determinants of the observed ability of several ginsenosides to compete with [^3^H]TCDD for binding to the gpAHR, PPD and PPT binding were computationally simulated. To this end, a homology model of the gpAHR LBD was developed using the procedure we previously described for other AHR LBDs [22], and ensemble docking calculations were performed for these ligands and TCDD (see Methods section for the details). The use of a homology model in lieu of an available experimental structure, along with the limitations of current docking methods in including protein flexibility during docking [34], prevented the use of the above computational protocol for modeling the binding of ginsenosides with larger molecular structures than PPD/PPT to the AhR.

**Figure 5:**
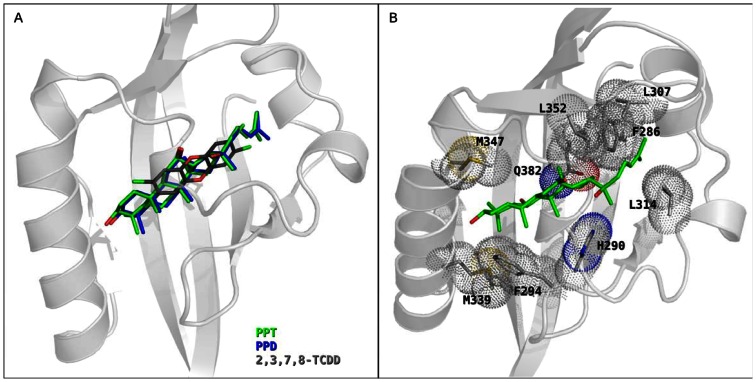
Ligand docking to the gpAHR LBD homology model. (A) Superimposition of the docking poses of PPT, PPD and TCDD (green, blue, and dark-gray sticks, respectively) in the gpAHR LBD model (cartoons). (B) PPT docking pose (green sticks) in the gpAHR LBD model (cartoons) with the most interesting interacting residues highlighted (light-gray sticks and van der Waals surfaces).

Docking calculations predicted a binding pose for the TCDD in the middle of the gpAHR binding cavity (see Figure 5A). Since this cavity shares similar structural characteristics and conserved internal residues with other mammalian AHRs with high TCDD affinity [35], the binding geometry was very similar to those predicted for mAHR and rtAHR [22]. Moreover, the TCDD∶gpAHR complex was stabilized by the same interactions previously predicted and validated by mutagenesis experiments of the TCDD∶mAHR complex [36], [37], [22].

Stable docking poses were obtained also for PPD and PPT within the binding cavity, with very similar placements (see Figure 5A). The interactions that mainly stabilized binding of these ginsenosides (Figure 5B) involved the central polar residues H290 and Q382, which interacted with the central hydroxyl groups of the ligands, as well as several hydrophobic residues at the entrance of the cavity (F294, M339 and M347) and lining the inner part of the cavity (F286, L307, L314 and L352). Some of these interactions were the same as those observed for TCDD. However, one difference was that TCDD is a relatively small molecule (228 Å^3^ volume), compared to PPD and PPT (389 Å^3^ and 390 Å^3^ volume, respectively) and in contrast to TCDD, the larger PPD and PPT molecules completely occupied the free internal space available in the LBD.

A confirmation of this precise fit between the ligand structures and the binding cavity features was supported by the results obtained by docking PPD and PPT to the mAHR, rtAHR and huAHR LBD models (data not shown). While docking poses similar to that described for gpAHR were obtained in the mAHR and rtAHR models, which share the same cavity features, no docking poses were obtained in the huAHR. There was a unique residue within the binding cavity of the huAHR (val381) that is different from that in the analogous position of the C57BL/6 mouse AHR LBD (ala375), and the lower affinity of TCDD for huAHR was attributed to the steric hindrance imparted by this residue that reduces the cavity space available, and affects TCDD placement and interactions [22], [35]. From our calculations, the presence of this residue in the modeled huAHR LBD, along with slight conformational differences of some hydrophobic residues in the inner part of the cavity, were sufficient to prevent PPD and PPT binding, although whether they actually interact with the huAHR remains to be determined. Finally, the ability of PPD and PPT to completely fill the available space in the gpAHR cavity (as well as in the mAHR and rtAHR cavities) could result in a severe reduction in the flexibility of the domain and a consequential inhibition of the conformational changes associated with ligand activation of AHR. This observation would be more consistent with the activity of PPD and PPT as TCDD antagonists than AHR agonists, which awaits experimental confirmation.

### Species Specificity Of Ginsenosides As Ahr Agonists In Rat, Mouse and Human Cell Lines

Dramatic species differences in the ability of chemicals to bind to and activate/inhibit the AHR have been previously reported [21], [38]. The above results indicate the ability of various ginsenosides to bind to and activate the gpAHR. Docking analysis indicate the ability of at least PPT and PPD to interact with the receptors from various species (although docking results with the huAHR could not be determined). Accordingly, to examine the species specificity of ginsenosides as AHR agonists, we evaluated their ability to induce DRE-luciferase gene expression in stably transfected rat (H4L1.1c4), mouse (H1L1.1c2) and human (HG2L6.1c3) hepatoma cell lines. Similar to the results obtained with guinea pig cells, most ginsenosides were inactive as AHR agonists. Rc is the only ginsenoside that consistently induced gene expression in guinea pig, rat and mouse cell lines (Figure 3 & Table 3). Rb_3_, Rc, Rh_1_, Rh_2_, F11 and TG induced gene expression in guinea pig cells; Rb_3_, Rc, Re, Rg_1_ and F11 induced gene expression in rat cells; Rc, Rg_1_ and PPT induced gene expression in mouse cells; Rb_2_, Rc and Rh_2_ induced gene expression in human cells.

### Dose-Dependent Induction Luciferase Activity of Rc In Different Cell Lines

**Figure 6:**
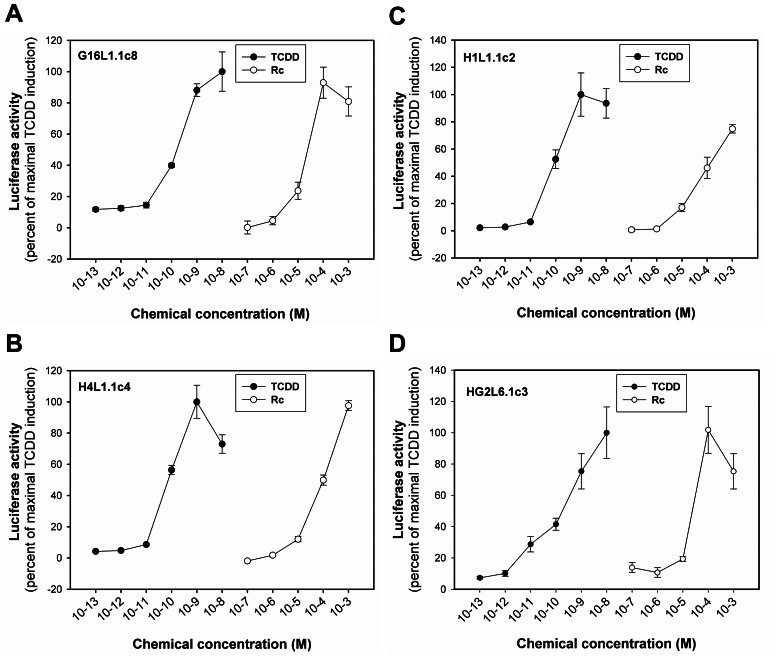
Dose-dependent induction of luciferase activity by TCDD and Rc in AHR-responsive recombinant guinea pig (G16L1.1c8), rat (H4L1.1c4), mouse (H1L1.1c2) and human (HG2L6.1c3) cells. Cells were incubated with the indicated concentration of Rc for 4 h and luciferase activity was determined as described in Materials and Methods section. Dose-dependent induction of luciferase activity by TCDD and Rc in (A) G16L1.1c8, (B) H4L1.1c4, (C) H1L1.1c2 and (D) HG2L6.1c3 cells were shown. Values were expressed in the figure as the percentage of maximal TCDD induction and represented the mean ± SD of triplicate determinations. All concentrations of TCDD ≥10^−11^ M and of Rc ≥10^−5^ M were significantly greater than DMSO-treated sample at p<0.01 as determined by Student's *t*-test.

Considering that Rc was the most efficacious ligand in the DNA and ligand binding assays, we chose Rc to examine the ability of different concentrations to induce luciferase reporter gene activity in cell lines from different species. Dose-dependent induction of luciferase by RC at 4 h was observed to levels greater than 75% of that induced by 1 nM TCDD in guinea pig, rat, mouse and human cell lines (Figure 6). The EC_50_ values of luciferase induction by Rc in G16L1.1c8, H4L1.1c4, H1L1.1c2 and HG2L6.1c3 cells were 11.5 µM, 100 µM, 127 µM and 13.3 µM, respectively. These results indicated that Rc was a relatively weak AHR agonist when compared to TCDD and other potent HAH and PAH ligands in these cell lines. Taken together, our results demonstrate the ability of selected ginsenosides to stimulate and/or inhibit the functionality of the AHR and AHR-dependent gene expression, with some compounds exhibiting species- and/or cell-specific differences in AHR responsiveness. Thus, ginsenosides represent a new class of naturally-occurring AHR ligands with compound selective agonist, antagonist or nonproductive ligand effects.

## Discussion

The AHR is a transcription factor that responds to structurally diverse ligands, and the activation of the AHR signal transduction pathway not only produces a spectrum of ligand-, species- and tissue-specific toxic and biological responses, but also plays a critical role in immune function, cardiovascular physiology and other endogenous functions [1]–[3]. Although TCDD and related toxic environmental contaminants have been extensively characterized as AHR ligands, an increasing number of reports have demonstrated the ability of a variety of endogenous chemicals and naturally occurring dietary compounds to interact with and activate the AHR and AHR signaling pathway. For example, numerous dietary phytochemicals have been shown to bind and/or modulate AHR action, and these include diindolylmethane, indole-3-carbinol (I3C) and its acidic condensation product indolo-[3,2b]carbazole, and various flavonoids, carotenoids and other chemicals [39]–[42]. In fact, recent studies have reported the ability of dietary AHR active compounds present in cruciferous vegetables to promote intestinal immune function, revealing the AHR as a critical link between diet and immunity [43], [44].

Here, we have demonstrated for the first time the ability of several naturally-occurring ginsenosides to directly bind to and activate/inhibit the AHR and AHR signal transduction pathway. Interestingly, our results indicate a divergence in response for these compounds, with several ginsenosides (e.g. Rc and Rh_1_) exhibiting pure AHR agonist activity, albeit as relatively weak agonists, while other compounds (e.g. Rh_2_, PPD and PPT) exhibited AHR antagonist activity, in that they could directly bind to the AHR, yet inhibited TCDD-dependent induction of AHR-responsive luciferase gene expression. In contrast, other ginsenosides like F11, the unique component found in American but not Asian ginseng species, exhibited novel AHR activity in that it could exert both agonist and antagonist activity at the same concentration. F11 could competitively bind to the AHR, stimulate AHR transformation, and DNA binding (agonist activity), but it also could inhibit TCDD induced AHR-dependent luciferase reporter gene expression (antagonist activity). While the mechanism of this novel action remains to be determined, this differential response could contribute to some of the differences in the biological and physiologic effects produced by American versus Asian ginseng species.

Comparison of the ability of each ginsenoside to induce AHR-dependent luciferase gene expression in cell lines from different species has revealed some interesting similarities and differences in species-specificity in response to these compounds. The ability of Rc to induce AHR-dependent gene expression to a comparable level in guinea pig, mouse, rat and human cell lines indicated that Rc was an AHR agonist of comparable potency/efficacy in each of these species. In contrast, comparison of luciferase gene induction in guinea pig, mouse, rat and human cells by all ginsenosides revealed numerous other ginsenosides exhibited species-/cell-specific differences in AHR agonist activity (Table 3). For example, ginsenoside Re induced luciferase to 38% of that of TCDD in rat H4L1.1c4 cells, yet little or no induction was observed in mouse, guinea pig or human cells. Similarly, Rh_1_ and TG induced luciferase activity in guinea pig cells to between 25–30% of that induced by TCDD, yet little or no induction was observed in other cells, while Rb_2_ could induce luciferase activity only in human cells to 19%. Rc induced luciferase activity in all the cell lines we used. While differences in cellular metabolism between these cell lines (i.e. differences in metabolic degradation of these compounds in different cell lines) could contribute to these species differences, species-specific differences in key amino acids within the ligand binding pocket of the AHR could also contribute to this differential response. In fact, comparison of the AHR ligand binding domain from different species revealed significant differences in amino acids within the ligand binding pocket [35], [37], and species-specific differences in AHR ligand binding specificity have previously been reported by several laboratories [10], [11]. While molecular docking approaches provided one avenue to examine specific interactions of ligands with amino acids within the modeled ligand binding pocket of the AHR from several species, current limitations as described above precluded our specific binding analysis of the majority of the ginsenosides. However, docking results with PPT and PPD not only revealed similarities in the specific binding interactions of these compounds within the gpAHR LBD, but also similarities in ligand binding characteristics between species. Whether species-specific binding interactions actually occur remains to be experimentally verified. In addition to ligand binding, the reported ability of various ginsenosides to affect cell signaling pathways and enzymes, coupled with the documented ability of some cell signaling pathways to affect AHR-dependent gene expression suggest that species- and/or cell-specific differences in these or other targets could contribute to the observed differences in AHR ligand efficacy and response [3], [45], although this remains to be confirmed. Taken together, these results confirmed AHR agonist activity of several ginsenosides in several species.

Comparing the relative activity of the ginsenosides in different AHR-based assays, some discrepancies in activity were apparent. For example, differences in the relatively efficacy/activity of Rc were observed in the guinea pig AHR bioassays. While Rc could stimulate AHR transformation and DNA binding to a level comparable to that of a maximal activating concentration of TCDD (Figure 3), AHR-dependent reporter gene activity was stimulated to only ∼24% of that of TCDD in the guinea pig cell line (Figure 2). Similar differences in AHR ligand efficacy/potency between *in vitro* and cell-based AHR assays have been previously observed and primarily resulted from the greater efficiency of a ligand to activate the cytosolic AHR *in vitro*, since the ligand has direct access to the AHR with little degrading enzymes present [46], [47]. A comparable response in intact cells would require all of the added ligand to enter the cell (without loss to serum proteins), it must avoid sequestration (by membranes, lipids, proteins, and organelles) and metabolism (by enzymes such as cytochrome P450s), and must find and bind to the AHR, fully stimulating AHR nuclear localization, transformation and DNA binding, and induction of gene expression, all within the time frame of the bioassays. Considering that ginsenosides appear to be highly susceptible to metabolism, significant reductions in their inducing potency in cell based assays are expected.

Major biological effects of ginsenosides include the enhancement of cholesterol biosynthesis [48], immunomodulatory and anti-inflammatory activity [49], antidiabetic and antioxidant effects [50], [51], cardiovascular protection [52], chemotherapeutic activities and neuroprotective effects [52], [53]. To date, more than 30 ginsenosides have been found in extracts of P. ginseng, with more than 60 isolated from members of the Panax genus, these include the oleanolic acid type ginsenosides (including R0), the 20 (S)-protopanaxadiol (PPD) type ginsenosides (including Ra, Rb, Rc, Rd, Rg_3_, Rh_2_ and Rs) and the 20 (S)-protopanaxatriol (PPT) type ginsenosides (including Re, Rf, Rg_1_, Rg_2_ and Rh_1_). While many of these compounds have shown to contribute to the wide range of medicinal effects of ginseng, it is likely that many distinct mechanisms of action (biological and toxic) exist for these different compounds, of which activation/inhibition of the AHR signaling pathway is only one. Considering the currently large consumption of ginseng and the diversity in effects, it is suggested that more extensive studies should be conducted on the biological and physiologic effects and mechanism of ginsenosides. Whether ginsenosides exert some of their beneficial clinical effects through an AHR-dependent pathway, and how exactly these structurally unique chemicals can bind within the AHR ligand binding pocket and activate/inhibit the AHR are exciting areas for future research.

## References

[1] Schmidt JV, Bradfield CA (1996) Ah receptor signaling pathways. Annu Rev Cell Dev Biol 12: 55–89. 8970722 10.1146/annurev.cellbio.12.1.55

[2] Safe S (1990) Polychlorinated biphenyls (PCBs), dibenzo-p-dioxins (PCDDs), dibenzofurans (PCDFs), and related compounds: environmental and mechanistic considerations which support the development of toxic equivalency factors (TEFs). Crit Rev Toxicol 21: 51–88. 2124811 10.3109/10408449009089873

[3] Denison MS, Soshilov AA, He G, DeGroot DE, Zhao B (2011) Exactly the same but different: promiscuity and diversity in the molecular mechanisms of action of the aryl hydrocarbon (dioxin) receptor. Toxicol Sci 124: 1–22. 21908767 10.1093/toxsci/kfr218 PMC3196658

[4] Kazlauskas A, Poellinger L, Pongratz I (1999) Evidence that the co-chaperone p23 regulates ligand responsiveness of the dioxin (Aryl hydrocarbon) receptor. J Biol Chem 274: 13519–13524. 10224120 10.1074/jbc.274.19.13519

[5] Meyer BK, Pray-Grant MG, Vanden Heuvel JP, Perdew GH (1998) Hepatitis B virus X-associated protein 2 is a subunit of the unliganded aryl hydrocarbon receptor core complex and exhibits transcriptional enhancer activity. Mol Cell Biol 18: 978–988. 9447995 10.1128/mcb.18.2.978 PMC108810

[6] Hankinson O (1995) The aryl hydrocarbon receptor complex. Annu Rev Pharmacol Toxicol 35: 307–340. 7598497 10.1146/annurev.pa.35.040195.001515

[7] Soshilov A, Denison MS (2011) Ligand displaces heat shock protein 90 from overlapping binding sites within the aryl hydrocarbon receptor ligand-binding domain. J of Biol Chem 286: 35275–35282. 21856752 10.1074/jbc.M111.246439 PMC3186366

[8] Denison MS, Seidel SD, Rogers WJ, Ziccardi M, Winter GM, et al.. (1998) Natural and synthetic ligands for the Ah receptor. Molecular Biology Approaches to Toxicology (Puga A, Kendall RJ, eds). London: Taylor and Francis, 3–33

[9] Ashida H, Fukuda I, Yamashita T, Kanazawa K (2000) Flavones and flavonols at dietary levels inhibit a transformation of aryl hydrocarbon receptor induced by dioxin. FEBS Lett 476: 213–217. 10913616 10.1016/s0014-5793(00)01730-0

[10] DeGroot D, He G, Fraccalvieri D, Bonati L, Pandini A, et al.. (2011) AHR ligands: promiscuity in binding and diversity in response. In: The Ah receptor in biology and toxicology, edited by Pohjanvirta R. 63–79, John Wiley & Sons, Inc. Hoboken, NJ.

[11] Denison MS, Nagy SR (2003) Activation of the aryl hydrocarbon receptor by structurally diverse exogenous and endogenous chemicals. Annu Rev Pharmacol Toxicol 43: 309–334. 12540743 10.1146/annurev.pharmtox.43.100901.135828

[12] Hestermann EV, Stegeman JJ, Hahn ME (2000) Relative contributions of affinity and intrinsic efficacy to aryl hydrocarbon receptor ligand potency. Toxicol Appl Pharmacol 168: 160–172. 11032772 10.1006/taap.2000.9026

[13] Huang KC, Williams WM (1999) The pharmacology of Chinese herbs. CRC.

[14] Tode T, Kikuchi Y, Kita T, Hirata J, Imaizumi E, et al (1993) Inhibitory effects by oral administration of ginsenoside Rh_2_ on the growth of human ovarian cancer cells in nude mice. Oncol. J Cancer Res Clin 120: 24–36. 10.1007/BF01200720 PMC12200171 8270603

[15] Wang Y, Ye X, Ma Z, Liang Q, Lu B, et al (2008) Induction of cytochrome P4501A1 expression by ginsenoside Rg_1_ and Rb_1_ in HepG2 cells. European journal of pharmacology 601: 73–78. 19022240 10.1016/j.ejphar.2008.10.057

[16] Delescluse C, Lemaire G, Sousa G, Rahmani R (2000) Is CYP1A1 induction always related to AHR signaling pathway? Toxicology 153: 73–82. 11090948 10.1016/s0300-483x(00)00305-x

[17] Hu W, Sorrentino C, Denison MS, Kolaja K, Fielden MR (2007) Induction of cyp1a1 is a nonspecific biomarker of aryl hydrocarbon receptor activation: results of large scale screening of pharmaceuticals and toxicants *in vivo* and *in vitro* . Mol Pharmacol 71: 1475–1486. 17327465 10.1124/mol.106.032748

[18] Vecchini F, Lenoir-Viale MC, Cathelineau C, Magdalou J, Bernard BA, et al (1994) Presence of a retinoid responsive element in the promoter region of the human cytochrome P4501A1 gene. Biochem Biophys Res Commun 201: 1205–1212. 8024563 10.1006/bbrc.1994.1833

[19] Garrison PM, Tullis K, Aarts JM, Brouwer A, Giesy JP, et al (1996) Species-specific recombinant cell lines as bioassay systems for the detection of 2,3,7,8-tetrachlorodibenzo-p-dioxin-like chemicals. Fundam Appl Toxicol 30: 194–203. 8812265 10.1006/faat.1996.0056

[20] Han D, Nagy SR, Denison MS (2004) Comparison of recombinant cell bioassays for the detection of Ah receptor agonists. Biofactors 20(1): 11–22. 15096657 10.1002/biof.5520200102

[21] Denison MS, Rogers JM, Rushing SR, Jones CL, Tetangco SC, et al.. (2002) Analysis of the Ah receptor signal transduction pathway, in: Current Protocols in Toxicology (Maines M, Costa LG, Reed DJ, Sassa S and Sipes IG, eds.). pp 4.8.1–4.8.45, John Wiley and Sons, NY. 10.1002/0471140856.tx0408s11 20945300

[22] Motto I, Bordogna A, Soshilov AA, Denison MS, Bonati L (2011) New Aryl Hydrocarbon Receptor Homology Model Targeted to Improve Docking Reliability. J Chem Inf Model 51: 2868–2881. 21981577 10.1021/ci2001617 PMC3263330

[23] Sali A, Blundell TL (1993) Comparative protein modeling by satisfaction of spatial restraints. J Mol Biol 234: 779–815. 8254673 10.1006/jmbi.1993.1626

[24] Marti-Renom MA, Stuart A, Fiser A, Sanchez R, Melo F, et al (2000) Comparative protein structure modeling of genes and genomes. Annu Rev Biophys Biomol Struct 29: 291–325. 10940251 10.1146/annurev.biophys.29.1.291

[25] Fiser A, Do RK, Sali A (2000) Modeling of loops in protein structures. Protein Sci 9: 1753–1773. 11045621 10.1110/ps.9.9.1753 PMC2144714

[26] Shen M, Sali A (2006) Statistical potential for assessment and prediction of protein structures. Protein Sci 15: 2507–2524. 17075131 10.1110/ps.062416606 PMC2242414

[27] MacroModel, version 9.9, Schrödinger, LLC: New York, NY, 2012.

[28] Maestro, version 9.3, Schrödinger, LLC: New York, NY, 2012.

[29] Kaminski GA, Friesner RA, Tirado-Rives J, Jorgensen WL (2001) Evaluation and Reparametrization of the OPLS-AA Force Field for Proteins via Comparison with Accurate Quantum Chemical Calculations on Peptides. J Phys Chem B 105: 6474–6487.

[30] Still WC, Tempczyk A, Hawlely RC, Hendrickson TA (1990) General treatment of solvation for molecular mechanics. J Am Chem Soc 112: 6127–6129.

[31] Glide, version 5.8, Schrödinger, LLC: New York, NY, 2012.

[32] Friesner RA, Murphy RB, Repasky MP, Frye LL, Greenwood JR, et al (2006) Extra precision Glide: Docking and scoring incorporating a model of hydrophobic enclosure for protein-ligand complexes. J Med Chem 49: 6177–6196. 17034125 10.1021/jm051256o

[33] Shigeyuki Uno, Kaori Endo, Yuji Ishida, Chise Tateno, Makoto Makishima, et al (2009) CYP1A1 and CYP1A2 expression: Comparing ‘humanized’ mouse lines and wild-type mice; comparing human and mouse hepatoma-derived cell lines. Toxicology and Applied Pharmacology 237: 119–126. 19285097 10.1016/j.taap.2009.03.001 PMC2752030

[34] Bordogna A, Pandini A, Bonati L (2011) Predicting the accuracy of protein-ligand docking on homology models. J Comput Chem 32: 81–98. 20607693 10.1002/jcc.21601 PMC3057020

[35] Fraccalvieri D, Soshilov AA, Karchner SI, Franks DG, Pandini A, et al (2013) Comparative analysis of homology models of the Ah receptor ligand binding domain: verification of structure-function predictions by site-directed mutagenesis of a nonfunctional receptor. Biochemistry 52: 714–725. 23286227 10.1021/bi301457f PMC3568667

[36] Pandini A, Denison MS, Song Y, Soshilov AA, Bonati L (2007) Structural and functional characterization of the Aryl hydrocarbon receptor ligand binding domain by homology modeling and mutational analysis. Biochemistry 46: 696–708. 17223691 10.1021/bi061460t PMC2860805

[37] Pandini A, Soshilov AA, Song Y, Zhao J, Bonati L, et al (2009) Detection of the TCDD binding fingerprint within the Ah receptor ligand binding domain by structurally driven mutagenesis and functional analysis, Biochemistry. 48: 5972–5983. 10.1021/bi900259z PMC2859071 19456125

[38] Denison MS, Vella LM, Okey AB (1986) Structure and function of the Ah receptor for 2,3,7,8-tetrachlorodibenzo-p-dioxin. Species difference in molecular properties of the receptors from mouse and rat hepatic cytosols. J Biol Chem 261: 3987–3995. 3005314

[39] Bjeldanes LF, Kim JY, Grose KR, Bartholomew JC, Bradfield CA (1991) Aromatic hydrocarbon responsiveness-receptor agonists generated from indole-3-carbinol *in vitro* and *in vivo*: comparisons with 2,3,7,8-tetrachlorodibenzo-p-dioxin. Proc Natl Acad Sci U S A 88: 9543–9547. 1658785 10.1073/pnas.88.21.9543 PMC52754

[40] Ciolino HP, Daschner PJ, Yeh GC (1998) Resveratrol inhibits transcription of CYP1A1 *in vitro* by preventing activation of the aryl hydrocarbon receptor. Cancer Res 58: 5707–5012. 9865727

[41] Henry EC, Gasiewicz TA (2003) Agonist but not antagonist ligands induce conformational change in the mouse aryl hydrocarbon receptor as detected by partial proteolysis. Mol Pharmacol 63: 392–400. 12527811 10.1124/mol.63.2.392

[42] Mukai R, Shirai Y, Saito N, Fukuda I, Nishiumi S, et al (2010) Suppression mechanisms of flavonoids on aryl hydrocarbon receptor-mediated signal transduction. Arch Biochem Biophys 501: 134–141. 20450880 10.1016/j.abb.2010.05.002

[43] Hooper LV (2011) You AHR what you eat: linking diet and immunity. Cell 147: 489–491. 22036556 10.1016/j.cell.2011.10.004

[44] Li Y, Innocentin S, Withers DR, Roberts NA, Gallagher AR, et al (2011) Exogenous stimuli maintain intraepithelial lymphocytes via aryl hydrocarbon receptor activation. Cell 147: 620–629. 10.1016/j.cell.2011.09.025 21999944

[45] Puga A, Ma C, Marlowe JL (2009) The aryl hydrocarbon receptor cross-talks with multiple signal transduction pathways. Biochem Pharmacol 77: 713–722. 18817753 10.1016/j.bcp.2008.08.031 PMC2657192

[46] Nagy SR, Sanborn JR, Hammock BD, Denison MS (2002) Development of a green fluorescent protein-based cell bioassay for the rapid and inexpensive detection and characterization of ah receptor agonists. Toxicol Sci 65: 200–210. 11812924 10.1093/toxsci/65.2.200

[47] Seidel SD, Li V, Winter GM, Rogers WJ, Martinez EI, et al (2000) Ah receptor-based chemical screening bioassays: application and limitations for the detection of Ah receptor agonists. Toxicol Sci 55: 107–115. 10788565 10.1093/toxsci/55.1.107

[48] Sakakibara K, Shibata Y, Higashi T, Sanada S, Shoji J (1975) Effect of ginseng saponins on cholesterol metabolism. I. The level and the synthesis of serum and liver cholesterol in rats treated with ginsenosides. Chem Pharm Bull (Tokyo) 23: 1009–1016. 1181066 10.1248/cpb.23.1009

[49] Joh EH, Lee IA, Jung IH, Kim DH (2011) Ginsenoside Rb_1_ and its metabolite compound K inhibit IRAK-1 activation-the key step of inflammation. Biochemical pharmacology 82: 278–286. 21600888 10.1016/j.bcp.2011.05.003

[50] Attele AS, Zhou YP, Xie JT, Wu JA, Zhang L, et al (2002) Antidiabetic effects of Panax ginseng berry extract and the identification of an effective component. Diabetes 51: 1851–1858. 12031973 10.2337/diabetes.51.6.1851

[51] Lee KT, Jung TW, Lee HJ, Kim SG, Shin YS, et al (2011) The antidiabetic effect of ginsenoside Rb_2_ via activation of AMPK. Archives of pharmacal research 34: 1201–1208. 21811928 10.1007/s12272-011-0719-6

[52] Chen X (1996) Cardiovascular protection by ginsenosides and their nitric oxide releasing action. Clinical and experimental pharmacology and physiology 23: 728–732. 8886498 10.1111/j.1440-1681.1996.tb01767.x

[53] Sato K, Mochizuki M, Saiki I, Yoo YC, Samukawa K, et al (1994) Inhibition of tumor angiogenesis and metastasis by a saponin of Panax ginseng, ginsenoside-Rb_2_ . Biological & pharmaceutical bulletin 17: 635–639. 7522731 10.1248/bpb.17.635

